# Drying methodology effect on the phenolic content, antioxidant activity of *Myrtus communis* L. leaves ethanol extracts and soybean oil oxidative stability

**DOI:** 10.1186/s13065-021-00753-2

**Published:** 2021-05-05

**Authors:** Ahmed Snoussi, Ismahen Essaidi, Hayet Ben Haj Koubaier, Houda Zrelli, Ibrahim Alsafari, Tesic Živoslav, Jelena Mihailovic, Muhummadh Khan, Abdelfatteh El Omri, Tanja Ćirković Veličković, Nabiha Bouzouita

**Affiliations:** 1 Higher School of Food Industries of Tunis (ESIAT), University of Carthage, 58 Avenue Alain Savary, 1003 Tunis El Khadra, Tunisia; 2 Laboratoire de Chimie Organique Structurale, Synthèse et Etude Physicochimique–Faculté des Sciences de Tunis, 2092 El Manar, Tunisia; 3 Institut Supérieur Agronomique de Chott Meriem, Université de Sousse, Sousse, Tunisia; 4 Genomics and Biotechnology Section, Department of Biological Sciences, Faculty of Science, King Abdulaziz University, Jeddah, Saudi Arabia; 5 Center of Excellence in Bionanoscience Research, King Abdulaziz University, Jeddah, Saudi Arabia; 6 Department of Chemistry, College of Science, University of Hafr AlBatin, Hafr Al Batin, Saudi Arabia; 7 Department of Biology, College of Science, University of Hafr AlBatin, Hafr Al Batin, Saudi Arabia; 8 Faculty of Chemistry, Belgrade, Serbia; 9 Center of Excellence for Molecular Food Sciences and Department of Biochemistry, Facult of Chemistry, University of Belgrade, Belgrade, Serbia; 10 Ghent University Global Campus, Incheon, South Korea; 11 Faculty of Bioscience Engineering, Ghent University, Ghent, Belgium; 12 Serbian Academy of Sciences and Arts, Belgrade, Serbia

**Keywords:** *Myrtus communis* L., Leaves, Drying methods, Phenolic compounds, Antioxidant activity, Soybean oil, Oxidation

## Abstract

In this study, different drying methodologies (convective air, oven and microwave) of *Myrtus communis* L. (*M. communis* L.) leaves were conducted to investigate their effects on the levels of phenolic compounds, antioxidant capacity of ethanolic extracts (EEs) as well as the soybean oil oxidative stability. Drying methodology significantly influenced the extractability of phenolic compounds. Microwave drying led to an increase in the amounts of total phenols, flavonoids and proanthocyanidins followed by oven drying at 70 °C. Higher temperature of drying (100 and 120 °C) led to a significant reduction of their amounts (p < 0.05). An ultra-performance liquid chromatography method combined with high resolution mass spectroscopic detection was used to analyze the phenolic fraction of extracts. Higher amounts of the identified compounds were observed when leaves were heat treated. Furthermore, the evaluation of the antioxidant activity showed that the studied extracts possess in general high antioxidant capacities, significantly dependent on the employed drying methodology. The incorporation of the different extracts at 200 ppm in soybean oil showed that its oxidative stability was significantly improved. Extracts from leaves treated with microwave (EE_MW) and at 70 °C (EE_70) have better effect than BHT. The results of the present study suggest that microwave drying could be useful to enhance the extractability of phenolic compounds and the antioxidant capacity of *M. communis* L. leaf extract.

## Introduction

Over the past few years, medicinal plants have attracted considerable attention as an efficient source of bioactive compounds, such as polyphenols endowed with antioxidant properties and could be used in the pharmaceutical, cosmetic and food industries [1]. Numerous epidemiological studies provided convincing evidence that consumption of foods or beverages rich in polyphenols led to the prevention of several life-style and chronic diseases such as cancer, obesity, cardiovascular and neurodegenerative diseases [2, 3]. The proposed mechanism of action of polyphenols was mainly related to an improved oxidative defense homeostasis. As a result, the demand for medicinal plants is increasing and strategies to promote their use in the abovementioned sectors will need to pay more attention to the factors that could impact the quantity and quality of phenolic compounds [4, 5].

Phenolic compounds extraction has several critical points like the choice of the plant phenological stage, the choice of solvent, the method of extraction and purification as well as the post-harvest technological processes mainly the drying process [6]. In fact, immediately after harvesting, fresh plants are usually dried in order to facilitate their transportation, storage and handling. Nevertheless, various chemical changes could occur during the drying affecting the content of phenolic compounds, thereby resulting in the change of the antioxidant activity [5]. Generally, a depletion of active principals and deterioration by reducing enzymatic activities are observed [7, 8]. As a consequence, the drying process is a key factor that enables to preserve the quality of medicinal plants and their bioactive extracts and compounds. Different methods are used to dry fresh plant tissues such as air drying, oven drying and microwave drying. Air and oven drying usually take a very long time which may lead to significant decrease of the quality of the dried product [9]. Microwave drying is an alternative way to improve the quality of dehydrated products. Microwave energy transfer causes a rapid evaporation of water from the plant tissue, treatment time is shorter, and quality is preserved [10, 11].

*Myrtus communis* L., belonging to the *Myrtaceae* family, is among the most representative medicinal plants of Tunisian flora, known also as myrtle. It is an evergreen shrub up to 3 m tall, growing mainly in humid and sub-humid bioclimatic stages. The fruits, leaves, roots and flowers are known for their medicinal, cosmetic, food and flavoring uses [12, 13]. The leaves are widely used in folk medicine for antiseptic, disinfectant, hypoglycemic agent, anti-gastric, appetizer, and antihemorrhagic purposes. It’s used for wound healing too where it’s applied externally [14, 15].

Myrtle is considered as an important source of phenolic compounds with several health benefits like antioxidant, antimicrobial and anti-viral activities [16, 17]. It is well-know that the amount of active secondary metabolites may vary depending on the plant organ, the season, the geographical area and the extraction technique. Myrtle leaves’ extracts were demonstrated to have the highest content in polyphenols as compared to flower buds and berries [18]. The active ingredients in myrtle can be classified into three main groups: (i) phenolic acids (Gallic acid, Syringic acid, Vanillic acid and Ferulic acid), (ii) flavonoids (Myricetin, Myricetin-3-*O*-galactoside, Myricetin-3-*O*-ramnoside, Quercetin, Quercetin-3-d-galactoside and Quercetin-3-d-rahmnoside), and (iii) tannins (Gallotannins and Catechin) [18].

A survey of the recent literature revealed that there was no report on the drying effect on *M. communis* L. leaves composition as well as in its use as food additive for lipid oxidation. Thus, the purposes of this work were i) to examine the extractability and the quality of phenolic compounds following different drying methodologies of *M. communis* L. leaves in correlation with its antioxidant capacity and ii) to evaluate the effectiveness of *M. communis* L. extracts on retarding the oxidation of soybean oil.

## Materials and methods

### Chemicals

Folin-Ciocalteu reagent, sodium carbonate anhydrous (Na_2_CO_3_), β-carotene, linoleic acid, 2,2-diphenyl-1-picrylhydrazyl (DPPH), 2,2′-azino-bis(3-ethylbenzothiazoline-6-sulphonic acid) radical cation (ABTS^.+^), 2,6-di-tert-butyl-4-methylphenol (butylated hydroxytoluene, BHT), aluminium chloride hexahydrate (AlCl_3_), hydrochloric acid (HCl), gallic acid, caffeic acid, quercetin, catechin were purchased from Sigma-Aldrich Chemie (Steinheim, Germany). Analytical grade ethanol, chloroform and Tween 40 were obtained from Merck (Darmstadt, Germany).

### Plant material

*Myrtus communis* L. leaves were collected from wild growing plants in the region of Ain Draham (North West of Tunisia). The botanical identification was performed according to the Tunisian flora [19]. Voucher specimens were deposited in the herbarium of the Higher School of Food Industries of Tunisia (ESIAT) for future reference. Our experiments were performed in compliance with our national guidelines wherever required.

### Drying methodologies

The drying procedures used in this study were optimized after several preliminary assays and based on a previous study conducted by Sellami et al. [20]. In fact, *M. communis* L. leaves were subjected to three different drying methodologies as fellow (i) Air drying process consisted of scattering myrtle leaves in trays in shadow at ambient temperature (16 °C) to be used as control, (ii) Oven drying carried out in ventilated oven at three different temperatures (70, 100 and 120 °C), and (iii) Microwave treatment was performed in a domestic digital microwave:500 g myrtle leaves, placed in glass beaker and heated at 500 W for 5 min. In case of microwave treatment, some preliminary experiments were conducted to evaluate the optimum treatment time at intermediate power (500 w). In fact, treatment for less than 5 min did not reduce the moisture content to a stable level which should be below 10%, and treatment for more than 5 min did significantly damage the sample.

### Ethanol extracts (EEs) preparation

Fifty grams of each sample were extracted separately by agitated maceration, at room temperature, with 80% aqueous ethanol for 72 h (3 × 300 mL), with changing solvent every 24 h. The obtained extracts were combined, filtered through a Whattman No.4 filter paper and concentrated under reduced pressure. EEs were stored at 4 °C until analysis.

### Determination of total phenols content

The Folin-Ciocalteu colorimetric method was adapted to estimate total phenols content, with gallic acid as a standard [21]. 100 *μ*L of the diluted extract (1/10 dilution) were mixed with 500 *μ*L of the Folin–Ciocalteureagent and 1 mL of distilled water. The obtained solution was stirred for 1 min then 1.5 mL of an aqueous solution of Na_2_CO_3_ (20%) was appended. The incubation of the mixture was conducted in the dark at room temperature for 120 min. The absorbance of all samples was measured at 760 nm and results were expressed in mg of gallic acid equivalents per gram (mg GAE/g) of dried extract.

### Determination of Total flavonoids content

Total flavonoids content estimation was realized with the AlCl_3_ method [22]. 1.5 mL of extracts was added to equal volumes of a solution of 2% AlCl_3_.6H_2_O. The mixture was thoroughly mixed and incubated for 10 min at room temperature; the absorbance was read at 367.5 nm. Data were expressed in mg quercetin equivalents per gram (mg QE/g) of dried extract.

### Determination of total proantocyanidins content

The acidic butanol technique was used to quantify proanthocyanidins content of the extracts [23]. 0.25 mL of extract was mixed with 3 mL of a 95% solution of n-Butanol/HCl (95:5 v/v) and 0.1 mL of NH_4_Fe(SO_4_)_2_. 12H_2_O solution in 2 M HCl. The prepared samples were incubated for 40 min at 95 °C. The absorbance was read at 550 nm against reagent blank. Data were expressed as mg catechin equivalents per gram (mg CE/g) of dried extract.

### LTQ Orbitrap LC–MS analysis

Myrtle ethanol extracts were subjected to LTQ Orbitrap LC–MS (Thermo Fisher scientific, Bremen, Germany) in order to profile, identify and quantify different phenolic compounds available in the extract, after drying the leaves at different temperature (room temperature, oven dried at 70, 100 and 120°C and microwave drying). The settings and details of the procedure were the same as our previous study [24]. Phenolic compounds were identified according to their spectral characteristics and retention time and the quantification was achieved using standard method as described by Boumaiza et al*.* [25].

### Free radical scavenging assays

The free radical-scavenging activity of EEs was determined through out two tests using the free radicals DPPH and ABTS^.+^.

#### DPPH radical scavenging activity assay

The assay was conducted as described by Chen et al. [26]. 2 mL of a serial extract concentrations in ethanol and 2 mL of ethanol for control sample were added to equal volume of a DPPH solution in ethanol (2.10^–4^ M) and incubated for 30 min in the dark at room temperature. The absorbance of the samples was measured at 517 nm using ethanol as a blank. The radical scavenging activity was expressed as IC_50_ (*μ*g/ml) which is the concentration providing the inhibition of 50% DPPH radicals. The inhibition percentage of free radicals was determined based on the equation: %inhibition = [1– AS/AC] × 100, where AC and AS are the absorbance of the control and the tested sample respectively at t = 30 min.

#### ABTS^.+^*radical scavenging activity assay*

The ABTS scavenging activity was evaluated following Re et al*.* [27] method with some modifications. The radical cation ABTS is generated by mixing equal volume 0.5 ml of a 3 mM potassium persulfate K_2_S_2_O_8_ solution and a solution stock of ABTS at 8 mM, protected from light at 4 °C for 16 h before use. The obtained solution is diluted with ethanol to reach an absorbance of 0.7 at 734 nm. 990µL of this freshly prepared solution is added to 10 µL of the EEs at different concentrations and the absorbance is read at 734 nm after 10 min.

The radical ABTS^·+^, in contact with a donor of H^·^ leads to the formation of ABTS in the solution which is discolored at 734 nm.

The inhibition percentage of ABTS^·+^ by the extract and the IC_50_ are given as described in the DPPH test.

#### β-Carotene/linoleic acid bleaching method

The antioxidant activity of EEs was estimated through β-carotene bleaching method as described by Suja et al*.* [28]. Briefly, 0.2 mg β-carotene, 20 mg linoleic acid and 200 mg Tween 40 were mixed in chloform. Then the mixture was concentrated by evaporating the solvent. The obtained residue was emulsified in 50 ml oxygenated distilled water and aliquoted. Finally, 0.2 mL of EEs (0.2 mg/ml), ethanol and BHT respectively for negative and positive controls were added to aliquots (4 mL) of the β-carotene/linoleic acid emulsion, and heated at 50 °C, in a water bath, in order to accelerate the oxidation. The absorbance was measured at 470 nm until the discoloration of the control solution (t = 120 min).

Antioxidant activity percentages (AA%) were calculated using the following equation:

$${\text{AA}}\% = \left[ {\left( {{\text{AS}}\left( {120} \right) - {\text{AC}}\left( {120} \right)} \right)/\left( {{\text{AC}}\left( 0 \right) - {\text{AC}}\left( {120} \right)} \right)} \right] \times 100$$

where, AS (120) is the absorbance of the tested sample at 120 min, AC (120) is the absorbance of the control at 120 min and AC (0) is the absorbance of the control at 0 min.

#### Effect of M. communis L. leaves extracts on soybean oil oxidative stability

##### Acidity

30 ml of neutralized ethyl alcohol are added to 5 g of oil to dissolve all the fat. The mixture is titrated with 0.17 N NaOH solution in the presence of phenolphthalein until a persistent light pink color is obtained at least 10 s.

##### Peroxide index

The peroxide index was determined based on spectrophotometric measurements at 560 nm of a mixture containing 300 mg oil samples, 9.9 ml choloroform/methanol mixture at a ration 7:3, 50 ml xylenol orange (10 mM). The mixture was catalyzed by iron (II) chloride, allowed to react for 5 min at room temperature, and then centrifuged at 1000*g* for 5 min. Results were expressed in milliequivalents of active oxygen per kg of oil.

##### Color

A tristimulus analysis of the color of the samples was carried out using a liquid colorimeter LOVIBONDPFX195 whose principle is based on the measurement of the percentage of the transmittance throughout the visible spectrum.

#### Statistical analysis

The data was processed using Statgraphics Centurion XVI for the determination of statistical significance as mean of triplicate ± standard deviation (mean ± SD). Further one-way ANOVA and Tukey HSD post hoc test was performed (P values < 0.05).

## Results and discussion

Drying processes in each of these three methods were carried out until achieving a percentage moisture content of about 10%. In the cases of air drying and ventilated oven treatments, dried myrtle leaves color turned to light brown with a green tinge. In contrast, microwave treatment changed the leaf color to slightly dark green. The loss of green color is mainly due to the degradation of chlorophyllic pigments.

### Effect of drying processes on total phenols, flavonoids and proanthocyanidins contents

**Fig. 1:**
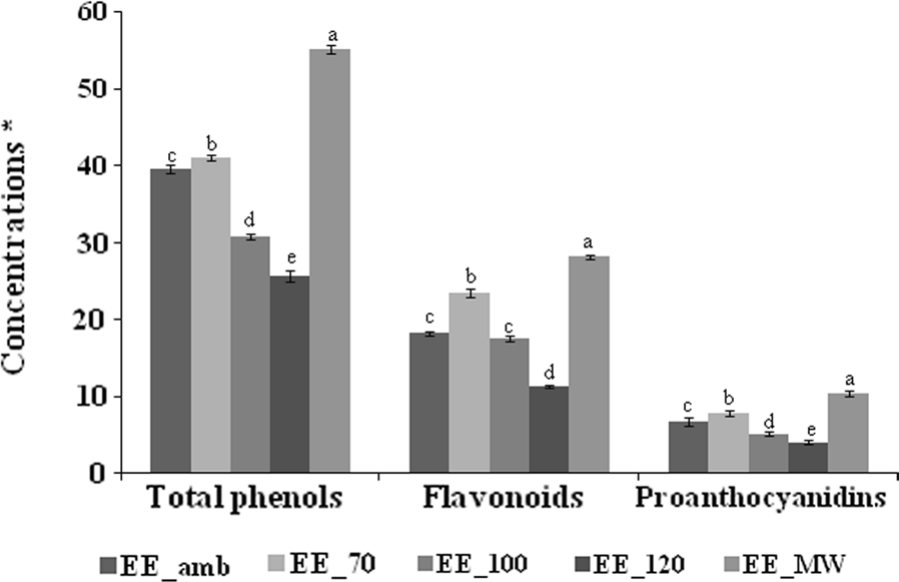
Total phenols, flavonoids and proanthocyanidins in ethanol extracts *M.communis* L. leaves subjected to different drying treatments. Total phenols were expressed in mg GAE/g, total flavonoids were expressed in mg QE/g, Total proanthocyanidins were expressed in mg CE/g. (EE_Tamb): leaves were dried at room temperature (16 °C); (EE_70, EE_100, and EE_120): leaves were oven dried at 70, 100, and 120 °C respectively; (EE_MW): leaves were dried in microwave as reported in Materials and Method. Each bar is the mean of 3 independent trials ± S.D. Data sharing different letter are significantly at p < 0.05 (one way ANOVA followed by Tukey Post hoc test)

Total phenols, flavonoids and proanthocyanidins contents of different extracts were significantly affected by the employed drying methodology (p < 0.05) (Fig. 1).

Total phenols in extracts increased from 39.6 GAE/g in air dried leaves to 55.2 GAE/g after drying with microwave and to 41.1 GAE/g in oven dried leaves at 70 °C. However, drying at 100  and 120 °C decreased the total phenol content to 30.8 and 25.7 GAE/g respectively.

As with total phenols, the amount of flavonoids in the extracts was dependent on the drying methodology ranging from 11.3 to 28.2 mg QE/g of the extract. Similarly, the extract from microwave dried leaves showed a higher flavonoids content (p < 0.05). As compared to air dried leaves, microwave drying increased 1.5 times the concentration of flavonoids in *M. communis* L. leaves extract.

Significant differences were also found in proanthocyanidins contents in the different studied extracts. EE_MW showed the highest content (10.5 mg CE/g).

### Identification and quantification of phenolic compounds LC–MS

We have previously shown that the LTQ OrbiTrap technique was proven to be reliable for the unambiguous detection of phenolic acids, their derivatives, and flavonoids based on their molecular masses and fragmentation pattern in unifloral Serbian honey samples [24].

**Fig. 2:**
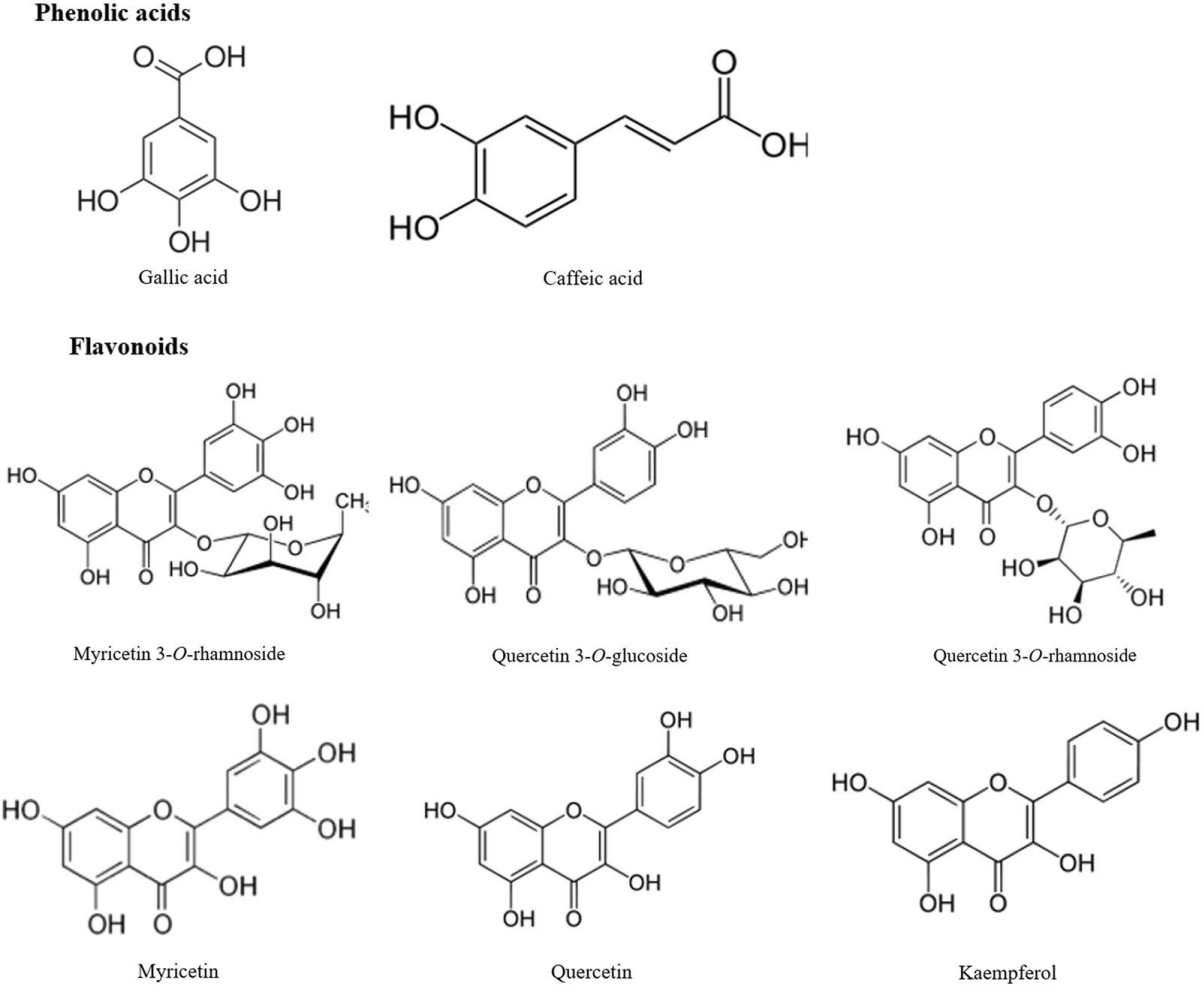
Chemical Structures of identified compounds in ethanol extracts of *M.communis* L. leaves subjected to different drying treatments

The chemical composition of different *M. communis* L. leaves extracts was analyzed using the LTQ Orbitrap LC–MS. This method provides the advantage to measure accurate mass data to four decimal places [29]. Eight phenolic compounds were identified in the different extracts including two phenolic acids: gallic and caffeic acids and six flavonoids: myricetin 3-*O*-rhamnoside, quercetin 3-*O-*glucoside, quercetin 3-*O*-rhamnoside, quercetin, myricetin and kaempferol (Fig. 2).

The employed drying methodologies affected significantly the amounts of the identified compounds in myrtle leaves extracts (Table 1).

**Table 1:** Contents (mg/g) of phenolic compounds in ethanol extracts of *M.communis* L. leaves subjected to different drying treatments

Phenolic compounds	Accurate mass [M – H]^−^	EE_Tamb	EE_70	EE_100	EE_120	EE_MW
Phenolic acids						
Gallic acid	169.0146	0.98 ± 0.06^b,c^	1.07 ± 0.14^b^	0.76 ± 0.04^c^	0.21 ± 0.03^d^	2.23 ± 0.11^a^
Caffeic acid	179.0351	0.18 ± 0.03^b^	0.21 ± 0.04^b^	0.14 ± 0.02^b^	0.14 ± 0.04^b^	0.35 ± 0.06^a^
Flavonoids						
Myricetin 3-*O*-rhamnoside	463.0891	0.46 ± 0.04^b^	0.49 ± 0.04^b^	0.37 ± 0.06^b^	0.24 ± 0.03^c^	0.71 ± 0.06^a^
Quercetin 3-*O*-glucoside	463.0955	0.15 ± 0.02^b^	0.16 ± 0.04^a,b^	0.11 ± 0.04^b^	0.08 ± 0.01^b^	0.24 ± 0.03^a^
Quercetin 3-*O*-rhamnoside	447.0769	0.23 ± 0.06^c^	0.42 ± 0.03^b^	ND	ND	0.67 ± 0.06^a^
Myricetin	317.0307	0.38 ± 0.07^c^	0.76 ± 0.06^b^	0.32 ± 0.06^c^	0.27 ± 0.05^c^	1.02 ± 0.02^a^
Quercetin	301.0357	0.42 ± 0.01^b^	0.48 ± 0.06^b^	0.31 ± 0.06^c^	0.12 ± 0.01^d^	0.77 ± 0.02^a^
Kaempferol	285.0407	0.12 ± 0.02^a^	0.14 ± 0.05^a^	0.11 ± 0.04^a^	0.09 ± 0.02^a^	0.16 ± 0.04^a^
	Total	2.12 ± 0.13^c^	2.87 ± 0.22^b^	1.50 ± 0.13^d^	1.08 ± 0.09^d^	4.27 ± 0.30^a^

HPLC profile was investigated as explained in Materials and Methods in Myrtle leaves ethanol extract subjected to different drying treatments. (EE_Tamb): leaves were dried at room temperature (16 °C); (EE_70, EE_100, and EE_120): leaves were oven dried at 70 °C, 100 °C, and 120 °C, respectively; (EE_MW): leaves were dried in microwave as reported in Materials and Method. Each value is the mean of 3 independent trials ± S.D. Data sharing different letter are significantly different at p < 0.05 (one-way ANOVA followed by Tukey Post hoc test)

ND: Not detected

The obtained results showed that microwave drying led to the highest increase in the amounts of the identified compounds (4.27 mg/g), two times higher than in air dried leaves extract (2.12 mg/g) and 1.5 times higher than in 70 °C dried extract (2.87 mg/g). Whereas, Oven drying at 100 and 120 °C led to significant reduction (p < 0.05) in their amounts. Drying at 100 °C caused a loss of 30% compared to air dried sample. The decrease was more pronounced when leaves were dried at 120 °C with a loss rate of 60%, leading to total content on phenolic compounds of 1.08 mg/g. Among the identified compounds, quercetin 3-*O*-rhamnoside was the most susceptible to high temperatures heat treatment. This compound was absent in oven drying leaves extracts at 100 and 120 °C.

The lack of available standards entailed us to limit the quantitative analyses to the mentioned compounds. Nevertheless, it is worthwhile to mention that *M. communis* L. leaves are rich in hydrolysable tannins as reported by Romani et al*.* [30], Yoshimura et al*.* [31] and Barboni et al*.*[32].

In the current study, gallic acid was the most abundant phenolic acid identified in myrtle leaves, which is concordant with previous finding of gallic acid distribution in different parts (leaf, stem and flower) of myrtle growing in Tunisia [16].

As regarding flavonoids, a class of secondary plant metabolites with powerful antioxidant activity [33], myricetin and myricetin-3-*O*-rhamnoside were most abundant compounds followed by the quercetin and its derivates. These results are in agreement with those reported by Barboni et al*.* [32] who studied the chemical composition of myrtle berry extracts by LC–MS/MS and HPLC–DAD and showed that myricetin and glycosides derivates were the major constituents.

The total phenolic compounds identified and quantified by LC–MS were significantly lower than that which were obtained by the Folin-Ciocalteu method which is a predictable method due to the weak selectivity of the reagent. It reacts with any reducing substance including phenolic and non-phenolic compounds [34].Although, there is a significant difference between the two methods, both of them demonstrated that the drying methodologies affected significantly the amounts of antioxidants in myrtle leaves extracts and indicated that the highest content of polyphenols was achieved in the case of microwave treatment.

### Effect of drying processes on the antioxidant activity

The EEs of heat treated *M. communis* L. leaves were evaluated for the antioxidant activity using the DPPH, the β-carotene bleaching method and ABTS^.+^ radical scavenging activity assay and compared to BHT. The antioxidant capacity is directly related to the content of phenolic compounds [5]. In this study, myrtle ethanolic extracts showed in general high antioxidant capacities significantly dependent on the employed drying methodology (Table 2).

**Table 2:** Antioxidant activities of ethanol extracts of *M.communis* L. leaves subjected to different drying treatments

	DPPH assay IC_50_ (µg/mL)	β-carotene method %AA	ABTS assay IC_50_ (µg/mL)
EE_Tamb	14.3 ± 0.4 ^d^	49.3 ± 1.0 ^d^	10.1 ± 0.6 ^d^
EE_70	7.6 ± 0.2 ^e^	60.7 ± 2.0 ^b^	5.2 ± 0.2 ^e^
EE_100	27.5 ± 0.7 ^b^	42.4 ± 0.7 ^e^	12.4 ± 0.9 ^b^
EE_120	48.5 ± 0.8 ^a^	34.7 ± 0.5 ^f^	41.0 ± 1.2 ^a^
EE_MW	2.4 ± 0.2 ^f^	68.1 ± 2.4 ^a^	2.7 ± 0.3 ^f^
BHT	20.0 ± 0.1 ^c^	58.2 ± 1.0 ^c^	10.7 ± 0.2 ^c^

(EE_Tamb): leaves were dried at room temperature (16 °C); (EE_70, EE_100, and EE_120): leaves were oven dried at 70, 100, and 120 °C, respectively; (EE_MW): leaves were dried in microwave as reported in Materials and Method. Each value is the mean of 3 independent trials ± S.D. Data sharing different letter are significantly different at p < 0.05 (one-way ANOVA followed by Tukey Post hoc test)

Though good the antioxidant activity of the extracts tested by the DPPH assay was basically dose dependent (data not shown). The extracts radical scavenging activity was determined by the amount of sample required for 50% scavenging of DPPH radical (IC_50_) which is inversely related to the antioxidant activity which means that higher IC_50_ the lower the antioxidant activity. IC_50_ ranged from 2.4 to 48.5 µg/ml and the differences of antioxidant capacities were very large up to 20-fold. The extract possessing the most powerful DPPH scavenging activity was obtained from microwave dried leaves with IC_50_ = 2.4 µg/ml. Concerning oven drying methodology, the highest effect was observed in the extract from 70 °C dried leaves (7.6 µg/ml), followed by the extract from 100 °C dried leaves (27.5* µg*/ml). Extract obtained with 120 °C dried leaves showed lower DPPH scavenging activity, indicating that higher temperature (> 100 °C) might inflict losses on the antioxidant activity [35].

Overall, *M. communis* L. leaves extracts showed higher DPPH scavenging activity than that of the synthetic antioxidant BHT (IC_50_ = 20 µg/ml). A negative correlation was observed between IC_50_ and total phenols (r = − 0.90, R^2^ = 81.90), flavonoids (r = − 0.93, R^2^ = 88.28) and proanthocyanidins (r = − 0.91, R^2^ = 83.48) which is in accordance with data we found in literature [34]. Where high phenolic content is often associated with similar increase in the scavenging capacity of the radicals [36].

For the β-carotene method, which evaluates the ability of plant extracts in inhibiting conjugated diene hydroperoxydes formation arising from linoleic acid oxidation, AA ranged from 34.7 to 68.1 and the difference of antioxidant activities were significant (p < 0.05). Nevertheless, all the tested extracts displayed intermediate antioxidant activities [37]. EEs from microwave and 70 °C heated leaves showed 68.1% and 60.7% inhibition, respectively, values higher than that of the synthetic antioxidant BHT (58.2%). The other extracts showed lower antioxidant activities. EE_120 showed the weakest antioxidant activity in the β-carotene method with an AA of 34.7% which was about the half of the activity achieved by EE_MW. A positive correlation was determined between AA and total phenols (r = 0.95, R^2^ = 91.66), flavonoids (r = 0.98, R^2^ = 96.28) and proanthocyanidins (r = 0.98, R^2^ = 96.31).

The free radical scavenging ability of the dried myrtle leaves extracts was also determined using the ABTS^.+^ radical cation. Based on the obtained results (Table 2) microwave dried leaves extract has the highest free radical scavenging ability and the order of ABTS^.+^results was consistent with that of DPPH and β-carotene assays. A negative correlation was observed between ABTS scavenging ability and total phenols (r = − 0.79, R^2^ = 62.14), flavonoids (r = − 0.88, R^2^ = 77.61) and proanthocyanidins (r = − 0.79, R^2^ = 63.29).

Despites a significant loss in their phenolic contents, EE_100 and EE_120 exhibited an antioxidant activity which could be due to the formation of new compounds having antioxidant properties during the drying procedure and the food processing [5, 38, 39].

Following the three drying processes, our results demonstrated that the microwave treated leaves were endowed with highest antioxidant activity. Such activity was almost three fold higher than that of the synthetic antioxidant BHT (IC_50_ = 20 µg/ml; AA = 58.2% and IC_50_ = 10.7* µg*/ml).

### Effect of *M. communis* L. leaves extracts on soybean oil oxidative stability

#### Effect of *M. communis* L. leaves extracts on soybean oil Acidity

**Fig. 3:**
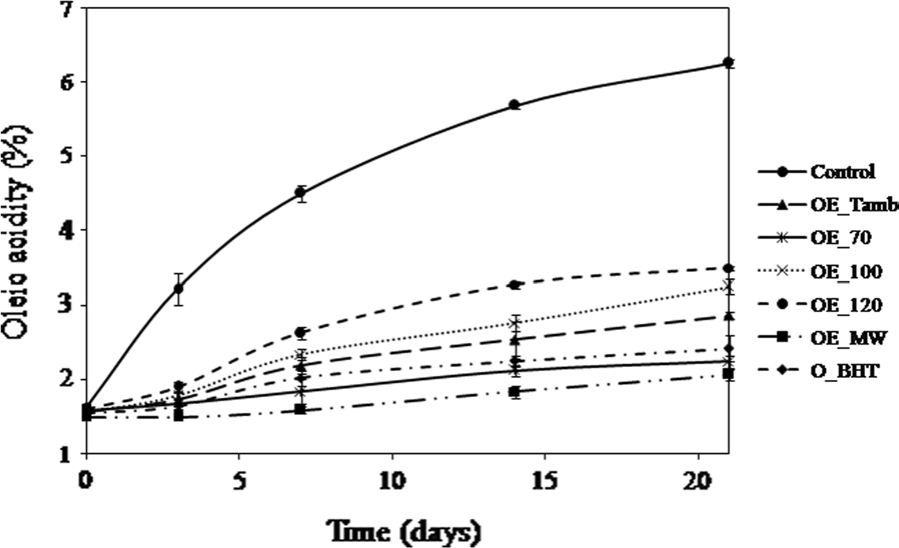
Evolution of oleic acidity (%) of soybean oil added with different extracts of *M.communis* L. leaves and BHT. Control: oil without antioxidant; (OE_Tamb): oil added with leaves extract dried at room temperature (16 °C); (OE_70, OE_100, and OE_120): oils added with extract of oven dried leaves at 70, 100 and 120 °C, respectively; (OE_MW): oil added with leaves extract dried in microwave; O_BHT: oil added with BHT. Each value is the mean of 3 independent trials ± S.D

The evolution of oleic acidity of soybean oil samples supplemented with extracts and BHT during storage for 21 days is shown in Fig. 3.

Acidity is the quality parameter of the oil that measures the degree of triglyceride hydrolysis. During the storage, an increase in of the acidity of all the oil samples was observed, however this increase is less accentuated in oils supplemented with *M. communis* L extracts and BHT. The increase in acidity is limited to 1.4 and 1.6 times respectively for the oil with EE_MW and BHT, whereas it is 4 times for the control sample.

The alterations leading to hydrolytic rancidity are enzymatic lipolysis and spontaneous hydrolysis of lipids. They are characterized by a sharp increase in the acidity of altered products [40].

#### Effect of *M. communis* L. leaves extracts on soybean oil Peroxide Value

**Fig. 4:**
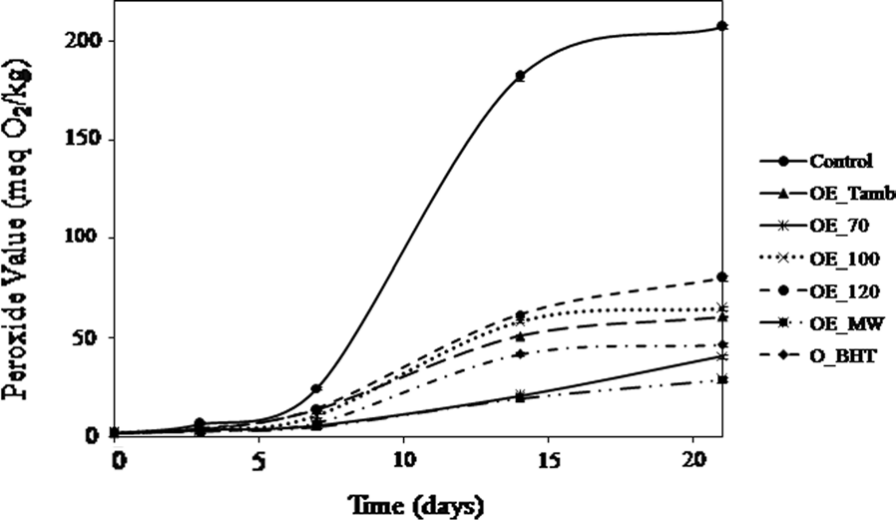
Evolution of peroxide values (meq O_2_/Kg) of soybean oil added with different extracts of *M.communis* L. leaves and BHT. Control: oil without antioxidant; (OE_Tamb): oil added with leaves extract dried at room temperature (16 °C); (OE_70, OE_100, and OE_120): oils added with extract of oven dried leaves at 70, 100 and 120 °C, respectively; (OE_MW): oil added with leaves extract dried in microwave; O_BHT: oil added with BHT. Each value is the mean of 3 independent trials ± S.D

The peroxide value is concerned with the number of active oxygens in the organic chains of a fatty substance (lipids, free fatty acids, monoglycerides, diglycerides and triglycerides). This index makes it possible to evaluate the oxidation degree of the unsaturated fatty acids of the fat (rancidity). The higher it is, the more the fat is oxidized. The results of the evolution of the peroxide values of the oil samples are illustrated in Fig. 4.

The oil samples have an initial peroxide value of the order of 2 meq O_2_/kg indicating the smooth running of oil production stages. During storage there is a rise in the peroxide value indicating the appearance of peroxides (primary oxidation products) in the oil samples. Synthetic antioxidant (BHT) oil samples and *M. communis* L extracts showed lower peroxide values than the control sample. After 21 days the peroxide value of the latter exceeds 200 meq O_2_/kg of oil which is 7xPV_OE_MW, 4.4xPV_OE_BHT. This variability in peroxide values can be explained by the difference in phenolic content, antioxidant potentials, and the ability to trap free radicals in soybean oil. The obtained results are correlated with those obtained for the in vitro study of the antioxidant activity of the extracts. The present findings agree with previous research who have reported the positive effect of rosemary extract on soybean oil oxidative stability [41]. The same results were obtained by Kamkar et al. [42] where the methanolic extract of *Mentha pulegium* was found to be able to preserve sunflower oil against oxidation.

However, this index is only an indicator of the beginning of oxidation. These phenomena are generally favored by high levels of unsaturated fatty acids which may constitute in some oils more than 80% of their mass, which is the case of soybean oil (48–59% linoleic acid, 17 to 30% oleic acid and 4.5 to 11% linolenic acid (Codex Alimentarius).

Indeed, exposed to air and light, all the oils develop, after a more or less long time, a rancid odor. This oxidative rancidity is due to the fixation of the oxygen of the air on the unsaturated fatty chains by a radical and auto-catalytic mechanism which results in the formation of hydroperoxides. These compounds are unstable and evolve, following complex mechanisms and still poorly known, to low molecular weight products responsible for the rancid odor. This phenomenon is accelerated by exposure to light, by the rise in temperature and the presence of metal catalysts. The rate of oxidation increases with the degree of unsaturation of fatty acids and is also influenced by the position of unsaturations within the fatty chain. The rancid odor depends not only on the degree of oxidation but also on the nature of the oxidized fatty acid (oleic, linoleic or linolenic acid). Each acid will lead to oxidation products of different structure and quantity [43].

#### Effect of *M. communis* L. leaves extracts on soybean oil Color parameters

The color of the oil may be an indication of its quality, the raw soybean oil good quality has a slight amber color which, after its neutralization, becomes a reddish-yellow color, and in the posterior deodorization, oil adopts the classic yellow color.

Monitoring the L*, of the soybean oil samples for 21 days gave the results summarized in Table 3.

**Table 3:** Evolution of the brightness parameter L* of soybean oil during storage

Samples	Storage time (days)				
	0	3	7	14	21
Control	95.66 ± 0.52^Aa^	93.34 ± 0.13^Ba^	93.06 ± 0.15^Ba^	89.40 ± 0.12^Cb^	87.31 ± 0.54^Dd^
OE_Tamb	91.44 ± 0.04^Ab^	90.54 ± 0.11^Bb^	89.55 ± 0.17^Cc^	88.58 ± 0.22^Dc^	87.14 ± 0.12^Ed^
OE_70	90.80 ± 0.20^Ab^	89.95 ± 0.06^Bc^	89.28 ± 0.35^Cc^	88.75 ± 0.15^Dc^	88.07 ± 0.05^Ec^
OE_100	90.17 ± 0.05^Ac^	89.11 ± 0.09^ Bd^	88.15 ± 0.08^Cd^	86.71 ± 0.08^Dd^	85.58 ± 0.05^Ee^
OE_120	90.89 ± 0.14^Ab^	88.87 ± 0.09^ Bd^	87.77 ± 0.20^Cd^	86.64 ± 0.26^Dd^	85.06 ± 0.05^Ee^
OE_MW	90.97 ± 0.13^Ab^	90.66 ± 0.24^Ab^	90.09 ± 0.07^Bb^	89.49 ± 0.07^Cb^	88.96 ± 0.06^Db^
O_BHT	95.73 ± 0.28^Aa^	94.11 ± 0.07^Ba^	93.51 ± 0.10^Ca^	93.22 ± 0.12^Ca^	92.64 ± 0.08^Da^

Control: oil without antioxidant; (OE_Tamb): oil added with leaves extract dried at room temperature (16 °C); (OE_70, OE_100, and OE_120): oils added with extract of oven dried leaves at  70, 100 and 120 °C, respectively; (OE_MW): oil added with leaves extract dried in microwave; O_BHT: oil added with BHT. Each value is the mean of 3 independent trials ± S.D

Mean values in the same row for each treatment followed by different lower-case superscript letters are significantly different (p < 0.05)

Mean values in the same line for each storage time followed by different upper-case superscript letters are significantly different (p < 0.05)

The initial values of L* reveal a difference between oil samples, in fact, a more apparent luminosity for the control samples and the BHT-added oil sample is observed where the L* values are 95.66 and 95.19 respectively. This difference is related to the dark color of the *M. communis* extract.

During storage, a decrease in the parameter L* is observed, this can be explained by a browning of the oil samples due to the oxidation phenomenon. The control oil sample shows the most apparent degradation of its lightness. The L* decreases by 8 units, whereas in the case of the oil containing EE_MW the decrease is only 2 units.

The presence of antioxidants that react with the free radicals, providing a stable form, and causing a transfer of the radical function to the antioxidant allows the maintenance of the oil to a more stable state and increases its shelf life and the preservation of its nutritional and organoleptic qualities [40].

Polyphenols, phytochemicals with diverse structural varieties, have gained recently greater attention because of some findings indicating their wide range of biological properties such as anti-cancer, antiviral and inhibition of oxidative mechanisms [44]. Their extraction process is complex and challenging as it depends on several factors such as the type of solvent, the solvent/solid ratio, the number of extraction steps, pH, time of contact, temperature as well as the post-harvest technological processes mainly the drying process. Until now, the majority of studies on myrtle have focused on the variability of essential oil and phenolic compounds among different plant parts [45, 46] and localities [32] or the effect of extraction method and solvent on the antiradical activity of myrtle leaf extracts [47]. To the best of our knowledge, there are no data about the effect of the drying methodology on the chemical composition or the antioxidant activity of myrtle leaves extract. Thus, the main objective of this manuscript was to increase awareness about the importance of this step which is often overlooked, not optimized, or not properly documented as sample preparation is often considered “as a means to an end” in most studies. In the present work, the obtained results demonstrate that drying process optimization is critical step for plant extracts preparation. In fact, plant extracts containing high antioxidant activities can be of multiple benefits for wellness and health maintenance and may increase the demand of these bioactive substances for food, cosmetic and pharmaceutical industries.

The beneficial effect of heat treatment on the antioxidant capacity could be attributed to an increase in phenolic compounds content [39]. According to Giusti et al. [48] the transformation of phenolic compounds from unextractable form to extractable form could be a plausible mechanism responsible for the elevated antioxidant activity of vegetal tissue during cooking. In the same way, Rakic et al*.* [49] found that hydrolysable tannins were degraded during thermal treatment of oak acorns leading to an increase of non-tannin phenolic content, especially gallic acid. As a result, thermal treatment lead to an increase in antioxidant activity when compared to the control. Accordingly, they suggest that the presence of gallic acid and its low molecular mass derivates caused the potent antioxidant properties of the studied samples [1].

Another possible explanation is the production of phenolic substances taking place during the drying. Indeed, the formation of additional compounds might occur due to the availability of precursors of phenolic compounds already present in the samples by non-enzymatic interconversion between existing polyphenols [50]. Nevertheless, high temperature (> 100 °C) treatment might destroy phenolic compounds of *M. communis* L. leaves. Our results are concordant with those reported by the literature. Indeed, similar results were also found by Ho and Lin [51] who reported that heat treatment of citrus peels at low temperature (< 100 °C) reduces the destructive impact of high temperature (150 °C) on the most abundant phenolic compound. As for Chan et al*.* [52], they observed, for *Thunbergia laurifolia* leaves, declines in total phenolic content (36%) and ascorbic acid equivalent antioxidant activity (25%) following oven-drying and enhancement by 38% and 84%, respectively after microwave drying compared to fresh leaves. The authors attributed the beneficial effect of microwave treatment on the antioxidant capacity to the rapid inactivation of polyphenoloxidase (PPO) activity in samples.

## Conclusion

In conclusion, the obtained results showed that *M. communis* L. leaves phenolic content and antioxidant activity are influenced by the drying methodology presenting the use of microwave as an alternative technique to enhance the phenolic compounds extractability and the antioxidant activity of *M. communis* L. leaves. The incorporation of the different *M. communis* L. leaves extracts have improved the oxidative stability of soybean oil. Therefore, the use of *M. communis* L. extract could be a promising way to prevent lipids oxidation in food.

## Data Availability

The datasets used and/or analysed during the current study are available from Dr Ahmed SNOUSSI on reasonable request.

## Abbreviations

*Myrtus communis* L.: *M. communis* L.
EE: Ethanol extract
Tamb: Ambient température
MW: Microwave
LTQ Orbitrap LC–MS: Liquid Chromatography Coupled with Linear Ion Trap Quadrupole Orbitrap Mass Spectrometry
LC–MS/MS: Liquid Chromatography Coupled with Tandem Mass Spectrometry
HPLC–DAD: High-performance liquid chromatography with a diode-array detector
mg GAE/g: mg of gallic acid equivalents per gram of dried extract
mg QE/g: mg quercetin equivalents per gram of dried extract
mg CE/g: mg catechin equivalents per gram of dried extract
DPPH: 2,2-Diphenyl-1-picrylhydrazyl
ABTS^.^^+^: 2,2′-Azino-bis(3-ethylbenzothiazoline-6-sulphonic acid) radical cation
IC_50_: Inhibition concentration at 50%
AA: Antioxidant activity
BHT: Butylated HydroxyToluene
N: Normality
PV: Peroxide value
meq O_2_/kg: Milliequivalents of active oxygen per kg of oil
PPO: Polyphenoloxidase
ND: Not detected
SD: Standard deviation
